# Adsorption Characteristics of Sodium Ions by Bentonite–Humic Acid Hydrogel: A Promising Water-Retaining Agent for Saline–Alkali Soil Improvement

**DOI:** 10.3390/gels11110927

**Published:** 2025-11-19

**Authors:** Weiye Liu, Mingjie Sun, Binghua Liu, Lin Peng, Xinghong Liu, Yanping Wang, Fangchun Liu, Hailin Ma

**Affiliations:** 1 Shandong Academy of Forestry Sciences, Jinan 250014, China; liuweiye5416@163.com (W.L.); mingjiesun1995@163.com (M.S.); binghualiu@163.com (B.L.); penglin0531@shandong.cn (L.P.); liuxh1969@126.com (X.L.); 2 College of Forestry, Shandong Agricultural University, Taian 271000, China; wangyp@sdau.edu.cn

**Keywords:** bentonite/humic acid hydrogel, microstructure, isotherm, kinetic, soil column

## Abstract

Sodium ions are the main harmful ions in coastal saline–alkali soils, and they seriously affect crop growth and soil structure. A bentonite/humic acid composite hydrogel, synthesized via graft copolymerization as a new type of water-retaining agent, can adsorb excessive Na^+^ in soil, thereby slowing down its adverse effects. This study used batch adsorption experiments to systematically investigate the effects of contact time, initial concentration, pH, temperature, and repeated cyclic adsorption on Na^+^ adsorption performance of the hydrogel material. The results indicated that Na^+^ equilibrium was achieved in 25 min, and the maximum adsorption capacity was 91.29 mg/g. Optimal adsorption occurred at pH 6–8.5, particularly in neutral to weakly alkaline conditions. At 30–50 °C, the bentonite substrate maintained excellent adsorption performance despite structural damage to the grafted copolymer. Mechanistic analysis revealed that adsorption followed pseudo-second-order kinetics and the Langmuir isotherm model, indicating chemisorption-dominated monolayer adsorption controlled by both intra-particle and liquid film diffusion. These findings demonstrate the potential of bentonite-based hydrogels for remediating coastal saline–alkali soils by mitigating Na^+^ toxicity.

## 1. Introduction

The Yellow River Delta in China is a newly formed land area shaped by land–sea interactions and the accumulation of large amounts of sediment carried by the Yellow River. However, high salinity seawater immersion levels and a high evaporation-to-precipitation ratio (3.5:1) mean that the soil in this region undergoes secondary salinization [1,2,3]. Sodium ions are the predominant cations in the groundwater, and Cl^–^ is the main anion. Together, they account for 65% of the total positive and negative ions, respectively [4]. Under strong evaporation conditions, salts in the groundwater rise to the surface through capillary action and accumulate after the water evaporates. Therefore, the salt content and composition of the groundwater directly influence the salt characteristics of the surface soil, resulting in Na^+^ and Cl^–^ being the dominant ions. The Na^+^ ion is a harmful ion in saline–alkali soils because it combines with soil colloids and reduces soil particle cohesion and permeability. This leads to the formation of a hardened layer that restricts water and air flow and damages soil structure [5]. Furthermore, Na^+^ accumulation disrupts the delicate ion balance in plants, which impairs their ability to absorb and utilize water and essential nutrients, as shown by previous studies [6].

The traditional restoration method for saline–alkali soil in the Yellow River Delta involves introducing fresh water from the Yellow River for salt pressing treatment. However, the scarcity of freshwater resources means that prolonged freshwater flooding can lead to resource wastage [7]. Bentonite-based hydrogels are widely used as soil amendments in arid regions and have gained attention in recent years because these gel materials have a strong water-holding capacity, improve soil structure, have slow nutrient release characteristics, are environmentally friendly, and are a relatively low-cost superabsorbent material [8,9]. Numerous studies have shown that bentonite-based hydrogels, when used as a soil amendment, can improve soil structure, increase field water-holding capacity, enhance soil nutrient levels, promote plant growth, and boost crop yields. The relative water content of soil increased by 30.7% when bentonite was applied at the ratio of 50 g/Kg to 30% FC soil under drought stress [10]. Kayama M observed that the application of bentonite in sandy soil in northeastern Thailand increased the retention rate of potassium in fertilizer by 32.9% [11]. After 7 years of single application of bentonite–humic acid, soil bulk density decreased by 9% at 0–10 cm, 10% at 10–20 cm, and 6% at 20–40 cm [12]. In Qingshuihe County, Hohhot, China, there were significant increases in soil microbial biomass carbon, biological nitrogen, and biological phosphorus. These improvements ranged from 5% to 50%, 3% to 42%, and 3% to 34%, respectively, when bentonite was added. They also reported that there was a significant improvement in soil quality, with soil organic matter, total nitrogen, and total phosphorus levels increasing by 1–15%, 1–19%, and 0–17%, respectively [13]. In the Binzhou field experiment, cotton yields increased by 3% to 20%, and water use efficiency improved by up to 29% after the application of bentonite [14]. The above studies focused on the effects of bentonite applications on soil properties and physiological and biochemical crop indices in arid regions. However, the Na^+^ adsorption mechanism is associated with bentonite-based hydrogel water-retaining agents (BHWAs). Their adsorption efficiency under different conditions and the effects on the gel’s morphological structure of the bentonite before and after adsorption are still not well understood. Therefore, this study investigated the Na^+^ adsorption characteristics and the structural changes in a bentonite/humic acid composite hydrogel. The aims of this study were to evaluate the feasibility of using hydrogel-based water-retaining agents to absorb water and control salt levels in saline–alkali soils and to explore the effects of soil salinity and moisture migration facilitated by these gel materials.

## 2. Results and Discussion

### 2.1. Effect of Adsorption Time

**Figure 1:**
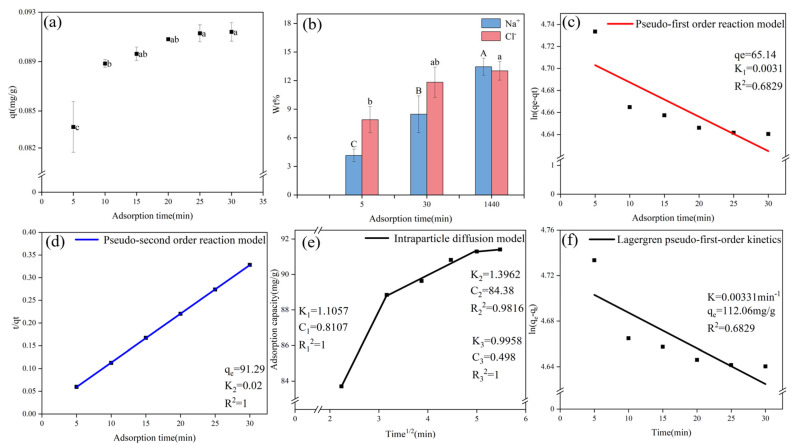
Image segmentation: (**a**) adsorption capacity of BHWA for Na^+^ under different adsorption times; (**b**) EDS weight percentage analysis of Na^+^ and Cl^−^ elements at different adsorption times; (**c**) quasi-first-order kinetic model; (**d**) pseudo-second-order kinetics model; (**e**) intra-particle diffusion model; (**f**) Lagergren pseudo-first-order kinetics model. Different letters above the error bars denote significant differences in means (*p* < 0.05, multiple comparisons).

The adsorption process can be divided into three stages (Figure 1a): the fast, slow, and equilibrium adsorption stages. During the initial stage (0–10 min), the adsorption capacity of the BHWA for Na^+^ significantly increased by 6.14% from 83.70 mg/g at 5 min to 88.84 mg/g at 10 min. The process then entered the slow adsorption stage (10–20 min), where the adsorption capacity significantly increased from 88.84 mg/g to 90.81 mg/g, which was a rise of 2.47%. However, the adsorption capacity did not significantly change during the last stage, which was the adsorption equilibrium stage (25–30 min).

**Figure 2:**
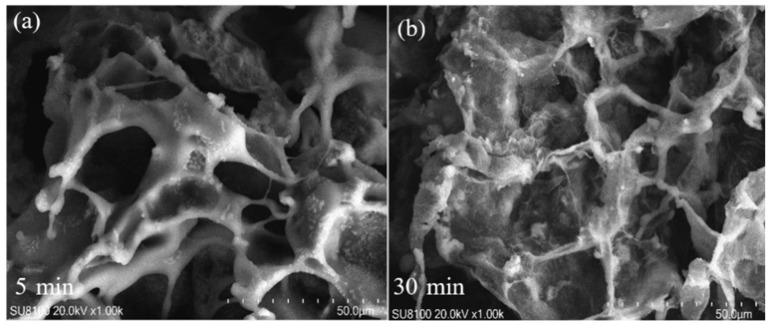
SEM images at different adsorption times: (**a**) SEM images of BMWA after adsorption in 9 g/L NaCl solution for 5 min; (**b**) SEM images of BMWA after adsorption in 9 g/L NaCl solution for 10 min.

In Figure 1, Different letters above the error bars denote significant differences in means (*p* < 0.05, multiple comparisons). The vertical bar means standard error. The network structure and voids in the bentonite hydrogel were clear and uniform after 5 min of adsorption (Figure 2). The pores were large, and the electric double layer was thick. When the adsorption time was extended to 30 min, then the pores became smaller and more uniform in size, the electric double layer had fully expanded, and its thickness became uniform. This change may be due to the bentonite hydrogel, the primary component of the water-retaining agent, undergoing structural modifications during the adsorption process.

Quasi-first-order and pseudo-second-order kinetic models were applied to the Na^+^ adsorption capacity results (Figure 1c,d). A comparison between the pseudo-first-order kinetic model and the pseudo-second-order kinetic model showed that the latter (qe = 91.46 mg/g, K_2_ = 0.02, R^2^ = 1) was the best-fitting model. The theoretical equilibrium adsorption capacity of the Lagergren model is 112.06 mg/g, and K = 0.00331 is small, which indicates that the adsorption process of BHWA is slow (Figure 1e). However, due to the R^2^ = 0.6829 of the model, the fitting degree of this model is poor, so it does not meet the quasi-first-order hypothesis. Therefore, the Na^+^ adsorption process associated with the BHWA was better described by the pseudo-second-order model [15]. This is consistent with other studies [16,17], suggesting that the adsorption process was primarily governed by chemical adsorption. In addition, the fitting parameters of the pseudo-second-order model suggested that the theoretical adsorption capacity of the BHWA for Na^+^ was 91.29 mg/g.

The maximum adsorption capacity of BHWA for Na^+^ (91.29 mg/g) exceeded that of silver tungstate-modified bentonite (Ag@OAB) for Rhodamine B (23.169 mg/g) reported by Sumardiono et al., demonstrating the effectiveness of the bentonite–humic acid composite structure for cation adsorption [18]. The superior performance of BHWA can be attributed to several factors: (1) the three-dimensional network structure increases porosity and accessibility of adsorption sites; (2) the carboxyl and hydroxyl groups from humic acid provide additional binding sites for cation adsorption; and (3) the composite material maintains the inherent cation exchange properties of bentonite while introducing new adsorption mechanisms through the grafted polymer network.

The pseudo-second-order kinetic model provided the best fit for BHWA (R^2^ = 1.0, k_2_ = 0.02 g/mg·min), which is consistent with the findings of Sumardiono et al., who also observed that Ag@OAB followed pseudo-second-order kinetics, indicating chemisorption as the primary mechanism. Similarly, the Langmuir isotherm model best described the adsorption behavior of BHWA (R^2^ = 0.991), suggesting monolayer adsorption on homogeneous surfaces, which aligns with the adsorption characteristics of similar bentonite composites [18].

The main solute transport mechanisms for solid–liquid adsorption processes are liquid film diffusion, intra-particle diffusion, or a combination of both. The adsorption rate of the adsorbent material is determined by the slowest step. Therefore, the rate-controlling step in the adsorption process is primarily affected by liquid film diffusion and intra-particle diffusion [19]. The intra-particle diffusion fitting curves for the BHWA used in this study were divided into three stages (Figure 1d), which suggested that intra-particle diffusion was not the only rate-controlling step during the adsorption of Na^+^ by the BHWA. After reaching the adsorption equilibrium, the Na^+^ content in the BHWA continued to increase, which confirmed this point (Figure 1b).

The K value reflects the diffusion rate of the adsorbate inside the adsorbent particles. During the adsorption process, K_1_ > K_2_ and R_1_ < R_2_, which suggested that intra-particle diffusion in the middle and late stages was not the main controlling factor. Instead, a diffusion process from the liquid film to the particles may have been the main process. There may also have been liquid film diffusion.

### 2.2. Effect of the Initial Solution Concentration on Adsorption

**Figure 3:**
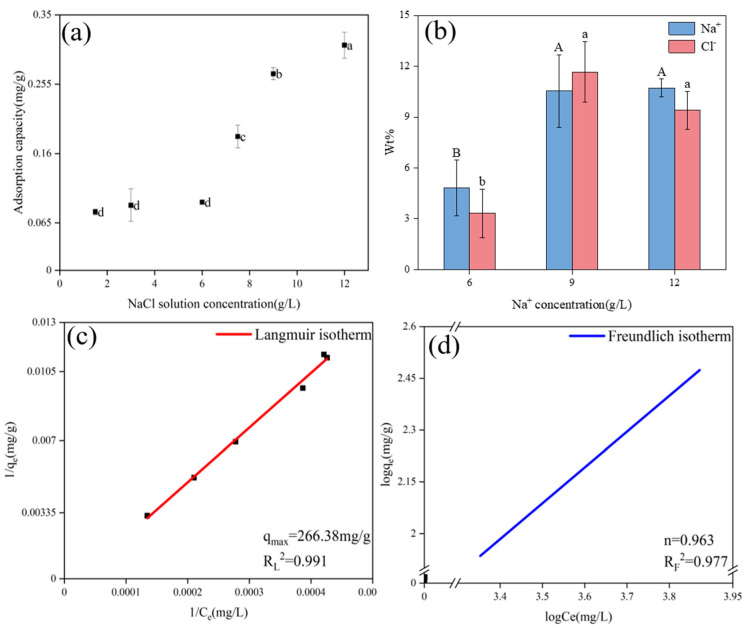
Image segmentation: (**a**) adsorption capacity of BHWA for Na^+^ at different solution concentrations; (**b**) EDS weight percentage analysis of Na^+^ and Cl^−^ elements at different solution concentrations (**c**) Langmuir isotherm model; (**d**) Freundlich isotherm model. Different letters above bars indicate statistical differences (*p* < 0.05) among treatments. Different letters above the error bars denote significant differences in means (*p* < 0.05, multiple comparisons).

**Figure 4:**
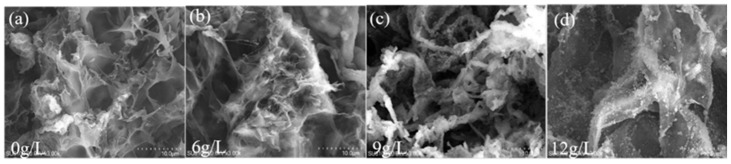
SEM images at different initial concentration solutions. (**a**) SEM images of BMWA adsorbed in deionized water for 24 h; (**b**) SEM images of BMWA adsorbed in 6 g/L NaCl solution for 24 h; (**c**) SEM images of BMWA adsorbed in 9 g/L NaCl solution for 24 h; (**d**) SEM images of BMWA adsorbed in 12 g/L NaCl solution for 24 h.

When the initial Na^+^ concentration was low (1.5 g/L–6 g/L), the adsorption capacity of the BHWA did not significantly increase as the concentration increased and the adsorption capacity remained at about 0.08–0.09 g/g (Figure 3a). However, when the Na^+^ concentration increased from 6 g/L to 12 g/L, the adsorption capacity increased from 0.09 to 0.31 g/g, which was a 244.44% increase, and significant differences were observed between each concentration. However, the EDS spectrum (Figure 3b) showed that when the solution concentration was 12 g/L, the Na^+^ and Cl^–^ concentrations in the BHWA were slightly lower than those in the 9 g/L solution, but there were no significant differences between the two solutions. Consequently, the BHWA that had been treated with deionized water and the 0 g/L, 6 g/L, 9 g/L and 12 g/L Na^+^ solutions were chosen for further characterization testing (Figure 4). The BHWA that had adsorbed pure water had a regular and continuous three-dimensional network structure with clear and evenly distributed pores. This suggested that in pure water, the water-retaining agent had swelled and stretched to its maximum extent and had formed a porous structure that promoted adsorption.

The network structure remained intact at the lower Na^+^ concentrations (6 g/L–9 g/L) (Figure 4), but swelling of the water-retaining agent was somewhat inhibited, and the three-dimensional network structure became disordered. The BHWA also agglomerated, which compromised structural integrity. The network structure was further altered in the 12 g/L NaCl solution. The agglomeration was more pronounced, and the original network structure was nearly unrecognizable. The SEM images showed no significant accumulation on the surface after adsorption, and the pores were largely filled. This indicated that the higher NaCl concentrations reduced swelling and disrupted the adsorption structure. The adsorption sites on the BHWA eventually became saturated [20].

Langmuir and Freundlich isotherm models were used to analyze the relationship between Na^+^ adsorption capacity and equilibrium state (Figure 3b,c). The Langmuir model assumes that ions are adsorbed on the surface in a monolayer. In contrast, the Freundlich model describes multi-layer adsorption at non-uniformly distributed surface sites. The correlation coefficients for the BHWA were R_L_^2^ = 0.991 and R_F_^2^ = 0.977, which shows that the Langmuir model was a better fit than the Freundlich model. Furthermore, the Langmuir model prediction of the saturated adsorption capacity was in good agreement with the experimental results. This indicated that Na^+^ adsorption by the BHWA was mainly monolayer adsorption [21]. Similarly, the adsorption of Congo red by the composite hydrogel with kaolin as the main component can also be accurately described by the Langmuir equation, which is related to the synergistic effect within the component [22].

The isothermal adsorption model also showed that Na^+^ adsorption was a monolayer adsorption process. Moreover, the reason why the BHWA had a large adsorption capacity and the internal ion content decreased at high concentrations may be that the higher Na^+^ concentrations provided a stronger ion exchange driving force. As a result, bentonite released more cations to exchange with Na^+^ in the solution, which led to a higher adsorption capacity at elevated concentrations [23,24]. However, the high-concentration solutions caused the structure of the BHWA to collapse. This reduced the ability of the Na^+^ to diffuse into the internal structure of the bentonite. Consequently, fewer adsorption sites were exposed on the surface, making it more difficult for Na^+^ to be adsorbed on the surface. Consequently, the Na^+^ migrated inward, which led to a decrease in the surface Na^+^ signal detected by EDS. However, this decrease was not significant, which suggested that the bentonite structure, the primary adsorbing component, had remained intact, whereas the three-dimensional network structure, based on graft copolymerization with humic acid as the skeleton, was disrupted. In the high ionic strength solutions, the swelling degree decreased, surface pores became denser, pore sizes decreased, and the electric double layer was compressed.

### 2.3. Effect of pH on Adsorption

**Figure 5:**
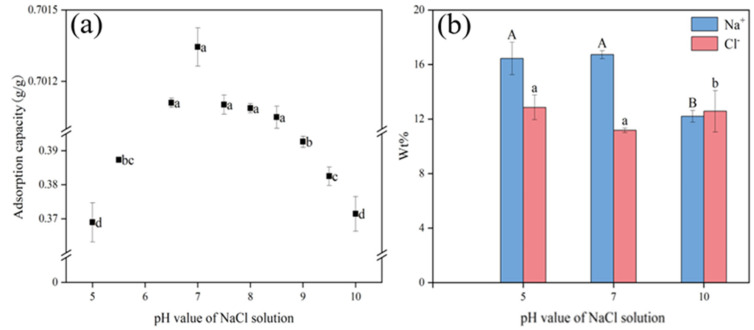
Image segmentation: (**a**) adsorption capacity of BHWA for Na^+^ at different pH; (**b**) %W of Na^+^ and Cl^−^ obtained by EDS analysis. Different letters above the error bars denote significant differences in means (*p* < 0.05, multiple comparisons).

The initial pH of the solution was set between 5 and 10 after taking into account the typical pH range under field conditions. Figure 5 shows that in acidic environments (pH 5–6), the Na^+^ adsorption capacity of BHWA was relatively low, ranging from 0.37 g/g to 0.39 g/g, and there was no significant difference between the pH treatments. When the BHWA was in neutral and weakly alkaline solutions (pH 6.5–8.5), its adsorption capacity was high and stable at 0.70 g/g, which was about 89.18% higher than that in acidic and alkaline environments. In alkaline environments (pH 8.5–10), the adsorption capacity decreased to 0.37 g/g, and there were significant differences between the treatments. The results indicated that changes in pH had a significant impact on the Na^+^ adsorption capacity of the BHWA. According to the EDS spectrum, there was no significant difference in Na^+^ content between neutral (pH 7) and acidic environments (pH 5), but it was significantly higher than that in alkaline (pH 10) environments (Figure 5b).

**Figure 6:**
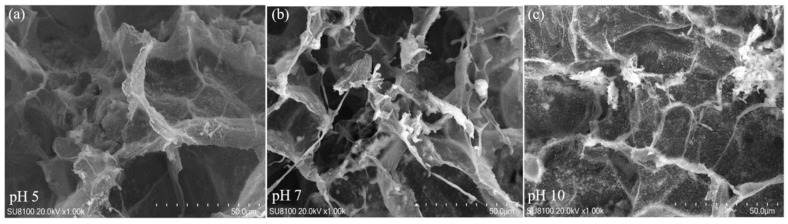
SEM images at different pH levels. (**a**) SEM images of BMWA adsorbed in 9 g/L NaCl solution at pH 5 for 24 h; (**b**) SEM images of BMWA adsorbed in 9 g/L NaCl solution at pH 7 for 24 h; (**c**) SEM images of BMWA adsorbed in 9 g/L NaCl solution at pH 10 for 24 h.

The morphology of the BHWA was characterized to observe its microstructure under acidic (pH 5), neutral (pH 7), and alkaline (pH 10) conditions (Figure 6). In the acidic environment (pH 5), the network structure became looser, although the gap distribution remained uniform. In the alkaline environment (pH 10), the thickness of the electric double layer decreased, the fibers became thinner and more scattered, and fiber breakage or agglomeration began to occur. Xiang et al. observed through TEM experiments that in 0.1 M NaOH solution (pH ≈ 13), the surface of montmorillonite showed signs of corrosion, and even some small corrosion pits were observed in 0.3 M (pH ≈ 13.5) and 1.0 M NaOH (pH ≈ 14) solutions [25,26]. Therefore, the decrease in the adsorbed Na^+^ and Cl^–^ contents under alkaline conditions was due to destruction of the bentonite structure. However, as the solution changed from acidic to neutral conditions (pH 5–8), the network structure remained intact, the pores were clear, and the pore distribution and thickness of the electric double layer were uniform. These factors enhanced the adsorption of Na^+^ by the BHWA, and the adsorption capacity increased as the pH of the solution rose from pH 5 to pH 8.

The adsorption capacity of the BHWA under acidic conditions (pH 5–6.5) was not significantly different from its adsorption capacity under alkaline conditions (pH 8.5–10), but the ion content under alkaline conditions was significantly lower than that under acidic conditions. The reason for this was that the high H^+^ levels under acidic conditions meant that the H^+^ competed with other cations in the solution for adsorption sites, which made less available for Na^+^. In contrast, the high concentration of OH^–^ under alkaline conditions dissolved the montmorillonite, which reduced the adsorption capacity of the BHWA.

The pH affects hydrolysis involving interlayer cations and alters the number and properties of the surface charge on the clay particles [27]. The silicon–oxygen tetrahedron structure in bentonite became negatively charged, while the aluminum–oxygen octahedron becomes positively charged. This may be due to the increased H^+^ concentration under acidic conditions. The higher H^+^ concentration in the solution neutralizes part of the negative charge on the bentonite, which enhanced the electrostatic effect [28]. This allows the bentonite colloid to easily adsorb ions and aggregate, which reduces the number of available adsorption sites. Under acidic conditions, the effective hydration radius of hydrated hydrogen ions (2.80 Å) is smaller than that of Na^+^ (3.58 Å). Therefore, hydrated hydrogen ions are more likely to adhere to the adsorption sites, which reduces the ability of the material to adsorb Na^+^ [29]. When the pH reaches 10, the bentonite becomes resistant to aggregation. This may be because the lower pH promoted the ionization of carbendazim cations and enhanced its adsorption by bentonite through the cation exchange process [30].

As the solution changed from neutral to alkaline, the OH^–^ concentration increased and the montmorillonite content gradually decreased. This change affected adsorption by bentonite and resulted in a decrease in its specific surface area, adsorption capacity, and expansion performance. The layered structure became uneven, surface roughness increased, and the adsorption capacity of the BHWA decreased [31]. These results were similar to those reported by previous studies [32].

The high OH^–^ concentration also increased the negative charge on the bentonite surface. This caused the electric double layer to expand, which should have enhanced the adsorption capacity. However, OH^–^ may have exchanged with the cations on the surface of the bentonite, which would increase resistance against aggregation. This would reduce the adsorption of other cations on the surface, thereby weakening the adsorption capacity of the BHWA [33]. The maximum adsorption of Na^+^ occurred when the pH of the solution was between 6 and 8.5. Goo showed that the structural group of the modified bentonite had cracks in it when the solution pH was 12.8, but there was no obvious desorption of Cs. This may be because the Cs retention capacity of bentonite was not significantly affected by the mineral and physicochemical changes to montmorillonite in alkaline and salt solutions [34]. The pHPZC of BHWA was determined to be 6.95. When the solution pH < pHPZC, the adsorbent surface carries a net positive charge, which electrostatically repels Na^+^ cations and reduces adsorption capacity. Conversely, when pH > pHPZC, the surface becomes negatively charged, favoring Na^+^ adsorption through electrostatic attraction. This explains the observed increase in adsorption capacity from pH 5 to pH 8. However, at pH > 8.5, despite favorable surface charge conditions, the adsorption capacity decreased due to montmorillonite dissolution under alkaline conditions, as evidenced by SEM analysis (Figure 6). Similar results were reported by Ye et al., who observed increased montmorillonite dissolution with rising pH using XRD analysis, leading to reduced swelling capacity of GMZ01 bentonite. Elevated temperatures further exacerbated this phenomenon [32].

### 2.4. Effect of Temperature on Adsorption

**Figure 7:**
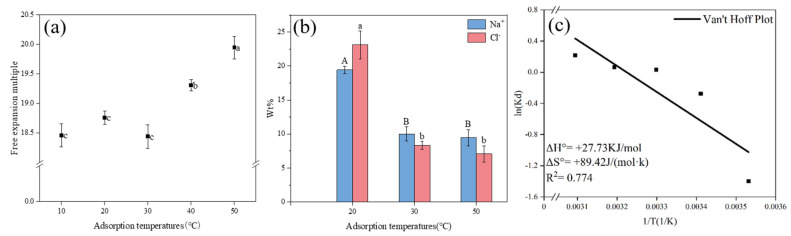
Image segmentation: (**a**) adsorption capacity of BHWA for Na^+^ at different temperatures; (**b**) %W of Na^+^ and Cl^−^ obtained by EDS analysis; (**c**) van ‘t Hoff plot. Different letters above the error bars denote significant differences in means (*p* < 0.05, multiple comparisons).

The expansion ratios of the BHWA were 18.46 g/g, 18.76 g/g, and 18.44 g/g when the temperatures ranged from 10 to 30 °C, but the differences were not significant (Figure 7a). The weight ratio for Na^+^ was 19.44% after adsorption in a 20 °C salt solution (Figure 7b). However, the free expansion rate of the BHWA increased sharply from 18.44 to 19.95 when the temperature rose from 30 °C to 50 °C, and the free expansion rate for the BHWA increased by 8.19% at 50 °C compared to its rate at 30 °C. The experimental data and analysis indicated that temperature significantly influenced the free expansion index, particularly beyond 30 °C, where incremental temperature rises led to a more pronounced effect on expansion behavior.

**Figure 8:**
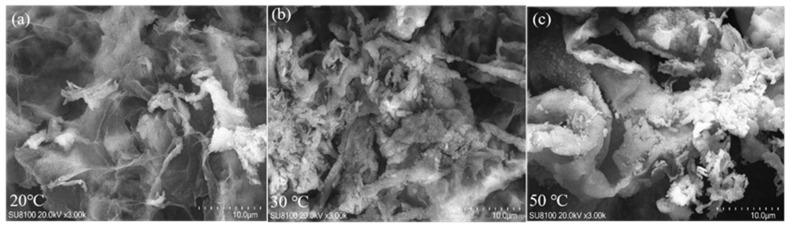
SEM images at different temperatures. (**a**) SEM images of BMWA adsorbed in 9 g/L NaCl solution at 20 °C for 24 h; (**b**) SEM images of BMWA adsorbed in 9 g/L NaCl solution at 30 °C for 24 h; (**c**) SEM images of BMWA adsorbed in 9 g/L NaCl solution at 50 °C for 24 h.

The BHWA in the different environments was scanned by an electron microscope, and its microstructure picture was obtained (Figure 8). At 20 °C, the BHWA still retained much of its pore structure, although the pores were relatively narrow with some adhesion observed (Figure 8a). At 30 °C (Figure 8b), the network structure began to collapse, the pore sizes and numbers were significantly smaller, and NaCl crystallization became more pronounced, with some pores being covered by the crystals. When the temperature reached 50 °C (Figure 8c), there was pronounced agglomeration, and the wrinkled, sparse porous network nearly disintegrated. These results agreed with other research, which showed that the maximum swelling strain for bentonite increased with temperature because osmotic swelling became the dominant factor [35].

The entropy of ions was temperature-dependent. At low temperatures, water molecules can enter the interlayer structure through Brownian motion, and the number that enters is based on the inherent adsorption capacity of the BHWA. In this study, the kinetic energy of the hydration shell decreased, which reduced the randomness of the water molecules within the hydration shell. These results were consistent with previous studies [27]. However, Papry found that the equilibrium adsorption capacity of bentonite increased slightly when the temperature rose from 10 to 40 °C, which was inconsistent with the results from this study [36]. The reason for this may be that the thermal kinetic energy of water molecules and Na^+^ also increased as the temperature increased. The hydration of Na^+^ led to the expansion of the inner layer spacing of the structure due to structural changes, which would decrease the number of available adsorption sites [37,38]. As a result, although the expansion ratio increased with temperature, the adsorption capacity decreased.

Thermodynamic analysis based on Gibbs free energy (Table 1) demonstrated that adsorption spontaneity was strongly temperature-dependent. At 10–20 °C, positive ΔG° values (+3.29 to +0.67 kJ/mol) indicate that the adsorption is thermodynamically unfavorable and non-spontaneous. Conversely, at 30–50 °C, negative ΔG° values (−0.08 to −0.58 kJ/mol) suggest that adsorption becomes spontaneous and thermodynamically favorable. The observed trend—whereby increasing temperature promotes spontaneity—is characteristic of endothermic adsorption processes [39]. Nevertheless, the van ‘t Hoff analysis (Figure 7c) yielded insufficient linearity (R^2^ = 0.774), indicating significant deviation from ideal thermodynamic behavior. Consequently, the thermodynamic parameters (ΔH° and ΔS°) derived from this plot lack reliability and are not recommended for quantitative reference or mechanistic interpretation.

### 2.5. Effect on Adsorption Capacity After Repeated Adsorption–Desorption

**Figure 9:**
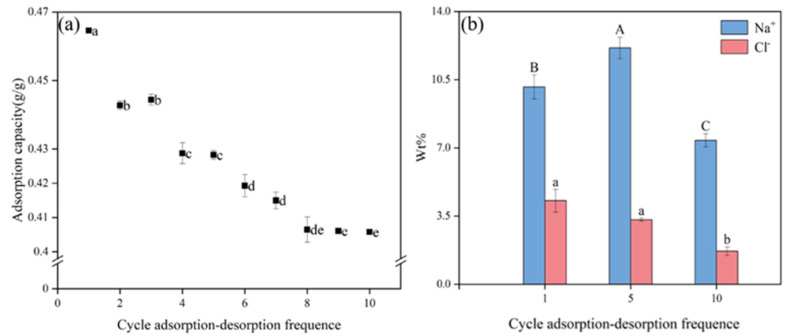
Image segmentation: (**a**) adsorption capacity of BHWA for Na^+^ under different adsorption–desorption frequencies; (**b**) %W of Na^+^ and Cl^−^ obtained by EDS analysis. Different letters above the error bars denote significant differences in means (*p* < 0.05, multiple comparisons).

The adsorption capacity during the 1st cycle was high at 0.4646 g/g (Figure 9a). However, it had fallen to 0.4065 g/g by the 8th cycle, which was a 12.51% decrease compared to the 1st cycle. Between the 8th and 10th cycles, the adsorption capacity decreased from 0.4065 g/g to 0.4058 g/g, which was 12.65% lower than that of the 1st cycle. However, the change trends for the Na^+^ and Cl^–^ contents in the BHWA were not the same. After the 1st adsorption–desorption cycle (Figure 9b), the Na^+^ content was 10.13%, which was lower than the 12.13% recorded after the 5th cycle but significantly higher than the 7.39% recorded after the 10th cycle. However, the Cl^–^ content decreased as the number of cycles increased, and the difference was significant after the 10th cycle, compared to 1st. The BHWA always maintained a large adsorption capacity for Na^+^ throughout the cycle, demonstrating that it has significant reusability.

**Figure 10:**
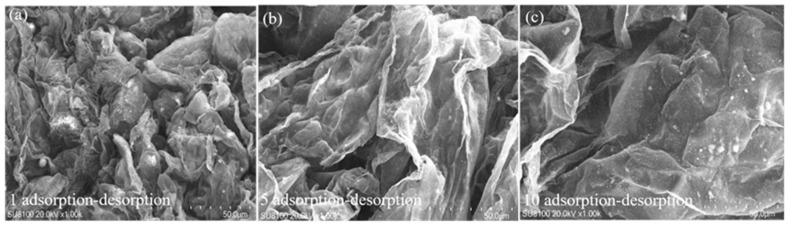
SEM at different adsorption–desorption frequencies. (**a**) The SEM image of BMWA after the first adsorption-desorption in 9 g/L NaCl solution was completed; (**b**) The SEM image of BMWA after the fifth adsorption-desorption in 9 g/L NaCl solution was completed; (**c**) The SEM image of BMWA after the tenth adsorption-desorption in 9 g/L NaCl solution was completed.

The degree of folding had decreased by the 5th cycle, and and contraction of the electric double layer was inhibited by the expansion and dehydration process (Figure 10). However, by the 10th cycle, the electric double layer was almost fully expanded, but the overall network structure of the BHWA was severely damaged. These results were similar to those observed by Mohammad [40].

The decrease in Na^+^ adsorption with the increase in cycle times may have been caused by the damage to the bentonite network structure after multiple cycles. During the repeated swelling and shrinkage process, bentonite collapsed in 5.85 g/L NaCl solution (solid/liquid = 1:40); the skeleton formed by graft copolymerization breaks and collapses, leading to a reduction in the number of effective adsorption sites for Na^+^ [35].

### 2.6. Principal Component Analysis of Adsorption Capacity Under Different Adsorption Conditions

**Figure 11:**
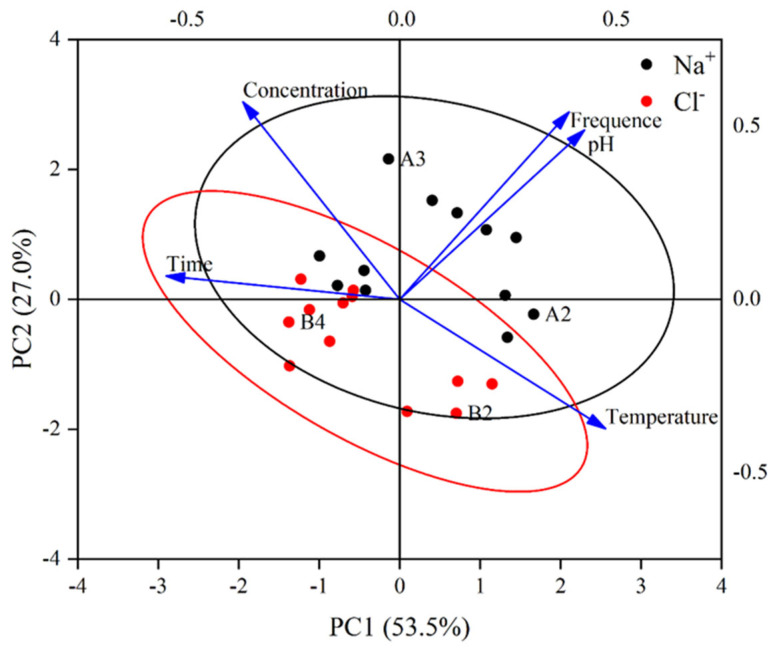
Principal component analysis. The black circles represent Na^+^, and the red circles represent Cl^−^. The black ellipse and red ellipse are the 95% confidence ellipses for Na^+^ and Cl^−^ groups, respectively, used to visualize the distribution and variation range of the two ions in the principal component space.

The first two principal components strongly represented the initial indicators (Figure 11). All the indicators had a higher load for the first principal component and were strongly correlated. The sodium and chlorine ion contents in the BHWA were analyzed using a principal component analysis (PCA). The PCA demonstrated that the first principal component (PC1) and the second principal component (PC2) accounted for 53.5% and 27.0% of the total variance, respectively, accounting for 80.487% of the total variance (Table 2). Temperature, pH, and frequency had strong positive loadings along PC1, whereas time and concentration had negative loadings. In contrast, concentration positively contributed to PC2, whereas temperature made a negative contribution. The directions and lengths of the variable arrows indicated that temperature, pH, and frequency were the main positive drivers of the sample distribution, whereas time and concentration had the opposite effects.

The spatial distribution of the samples showed that the Na^+^ samples were mainly located in regions associated with higher temperatures, pH, and frequency, whereas the Cl^−^ samples tended to cluster in areas characterized by larger time and concentration values. The distinct 95% confidence ellipses for Na^+^ and Cl^−^ suggest that the environmental and chemical factors led to statistical differences in ion adsorption by the bentonite. This separation implied that Na^+^ and Cl^−^ responded differently to environmental conditions, possibly due to differences in migration pathways or sensitivity to environmental change. The results suggested that increased temperature, pH, and frequency increased Na^+^ penetration of the bentonite interior, while longer timespans and higher concentrations were associated with Cl^−^ accumulation.

### 2.7. Effect of BHWA on Water and Salt Transport in Soil

**Figure 12:**
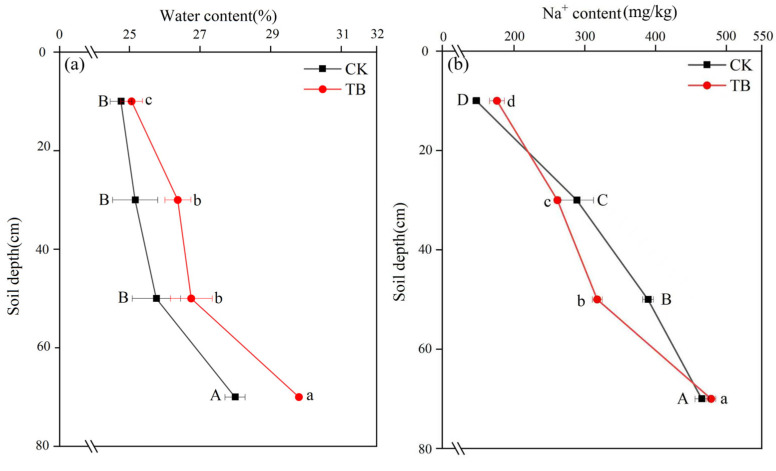
Image segmentation: (**a**) soil water content; (**b**) soil Na^+^ content. Different letters above the error bars denote significant differences in means (*p* < 0.05, multiple comparisons).

The CK water content decreased from 27.99% (60–80 cm) to 24.76% (0–20 cm) in the 0–80 cm soil layer, which was a decrease of 14.26%, and the TB water content decreased from 29.8 g/g (60–80 cm) to 25.06 g/g (0–20 cm), which was a decrease of 18.91% (Figure 12a). Therefore, the TB water content in the 0–80 cm soil layer was greater than that of CK. Compared to CK, the maximum increase of 6.47% occurred in the 60–80 cm layer, and the minimum increase of 1.21% occurred in the 0–20 cm soil layer. Ninthap found that the addition of bentonite increased soil water-holding capacity by 35%, which was consistent with the results from this study [41].

The Na^+^ contents were significantly different among the different soil layers in the same treatment (Figure 12b). The Na^+^ content in CK decreased from 470 mg/kg to 150 mg/kg from the top to the bottom of the 0–80 cm soil layer, which was a decrease of 68.09%. The Na^+^ content in TB decreased from 480 mg/kg to 180 mg/kg, a 62.50% decrease. In the 20–60 cm soil layer, the Na^+^ content in the TB treatment was lower than that of CK. Lerma reported that a bentonite-based modifier increased dry leaf weight by 1.9 times, which indicated that bentonite could reduce the degree of soil salinization, thus alleviating Na stress on plants [42]. This result was consistent with the results from this study.

The Na^+^ content in the TB treatment was greater than that of CK in the 0–20 cm and 60–80 cm soil layers. At the end of the experiment, the total water absorption by CK was 667 mL, whereas water absorption by TB was 847 mL, which was 26.99% higher than that of CK. Bajestani found that adding bentonite/alginate/nanocellulose composites to the soil reduced the net water flow by 75.30%, which was similar to the results from this study [43]. The water content in the TB treatment at the same depth was higher than that of CK. This meant that it had adsorbed the NaCl solution to a greater extent, resulting in a higher Na^+^ content. In general, most of the soil water in the 0–20 cm soil layer evaporates, leaving Na^+^ in the soil. In this study, the BHWA allowed the water to reach the surface of the soil more rapidly than in CK. This enabled it to evaporate more rapidly and led to an increased accumulation of Na^+^.

## 3. Conclusions

This study investigated the adsorption properties of a BHWA for Na^+^ under different conditions: water absorption time, initial NaCl concentrations, solution pH, and reaction temperatures. The Na^+^ adsorption process by BHWA conformed to the pseudo-second-order kinetic model. The adsorption of Na^+^ by BHWA was a fast process, and the adsorption equilibrium can be reached in 30 min. According to the pseudo-second-order kinetic equation, the theoretical maximum adsorption capacity of Na^+^ is 91.29 mg/g. However, under isothermal conditions, the adsorption process conformed to the Langmuir model, which indicated that the adsorption of Na^+^ by the BHWA was a chemical adsorption process with monolayer adsorption. The diffusion rate was governed by both intra-particle diffusion and liquid film diffusion, and the structure of the BHWA was not destroyed under acidic conditions. Thermodynamic analysis confirmed the endothermic nature of Na^+^ adsorption, with spontaneous adsorption occurring at temperatures above 30 °C, which is favorable for field applications. However, when the OH^–^ concentration (pH > 8.5) was high, the main component of the BHWA, montmorillonite, broke down and was dissolved in the solution. The results showed that BHWA increased soil water-holding capacity and reduced the degree of soil salinization by adsorbing Na^+^.

## 4. Materials and Methods

### 4.1. Materials

BHWA was prepared by graft copolymerization of bentonite, potassium humate, and acrylamide (mass ratio 8:1:3) in the presence of acrylic acid and thiourea. The pH was adjusted to 6–8, and polymerization was initiated with APS/NaHSO_3_ at 60 °C. The product was washed, dried, and sieved to 0.25–0.5 mm

The BWHA used in this study was a mineral-based material with a three-dimensional network structure. The BHWA exhibited a near-neutral pH of 6.95 when dispersed in deionized water (solid-to-liquid ratio of 0.25 g: 50 mL), suggesting negligible interference with the pH of experimental solutions during adsorption tests. It was created through graft copolymerization using layered Na–bentonite and humic acid as the skeleton, with bentonite comprising 40–60% of the material. Particles with a size range of 0.25–0.5 mm were selected to test the material. Sodium chloride (NaCl, molecular weight: 168.36 g/mol, purity: 99.99%) was dissolved in deionized water at various concentrations to prepare sodium (Na)-containing solutions. The pH of the salt solution was adjusted to the desired level using standard hydrochloric acid (HCl) and potassium hydroxide (KOH) solutions. A total of six 10 cm diameter plastic measuring cups and six transparent acrylic tubes, each 80 cm in length and with an inner diameter of 6 cm, were used in this experiment. Each tube had an opening at one end and a sieve plate at the other end. The soil used in the experiment was taken from the surface soil of the nursery at Shandong Academy of Forestry Sciences, China.

### 4.2. Experimental Scheme Design

#### 4.2.1. Kinetic Adsorption and Isothermal Adsorption

A total of 0.25 g of a synthesized super water-retaining agent based on bentonite that contained sulfonic acid and carboxylic acid groups was placed in 100 mL centrifuge tubes, and then 50 mL of 0.9% NaCl solution was added. The tubes were oscillated at 220 rpm and 25 °C, and samples were taken at regular intervals (0.5 h, 1 h, 2 h, 4 h, 8 h, 16 h, 24 h, and 48 h) to assess their water retention properties. The supernatant was filtered using a 0.45 µm water filter head, and the Na^+^ content was measured using a flame photometer. The BHWA was then removed, freeze-dried in a freeze-drying box, and stored for the characterization test. An adsorption test was used to determine the maximum Na^+^ removal rate by the water-retaining agent.

The adsorption pseudo-first-order and pseudo-second-order kinetics are as follows:

Quasi-first-order kinetic equation:

(1) $$\ln\left(q_{e}-q_{t}\right)=\ln q_{e}-\frac{K_{1}}{2.303}t$$

Pseudo-second-order kinetic equation:

(2) $$\frac{t}{q_{t}}=\frac{1}{K_{2}q_{e}^{2}}+\frac{1}{q_{e}}$$

Lagergren pseudo-first-order kinetics equation:

(3) $$ln\left(qe-qt\right)=lnqe-Kt$$

where q_t_ is the adsorption capacity (mg/g) of the adsorbent at time t; q_e_ is the equilibrium adsorption capacity of the adsorbent (mg/g); K_1_ is a pseudo-first-order adsorption rate constant; and K_2_ is a pseudo-first-order adsorption rate constant.

The isothermal adsorption equations are as follows:

Langmuir isothermal equation:

(4) $$\frac{C_{e}}{q_{e}}=q_{m}-\frac{q_{e}}{K_{L}C_{e}}$$

Freundlich isothermal equation:

(5) $$\log q_{e}=\log K_{F}+\frac{1}{n}\log C_{e}$$

where q_m_ is the theoretical saturated adsorption capacity; K_L_ is Langmuir constant, and K_F_ and n are Freundlich equation constants.

#### 4.2.2. Effect of Reaction Conditions (Initial pH Value of the Solution and Reaction Temperature) on the Adsorption Performance of the Water-Retaining Agent Test

A total of 0.25 g of the BHWA was placed into 50 mL centrifuge tubes to conduct two sets of experiments:

(1) Effect of the Initial Solution pH:

The pH of a 9 g/L NaCl solution was adjusted to 6, 6.5, 7, 7.5, 8, and 8.5 using HCl and KOH solutions. Then, 50 mL of each pH-adjusted solution was added to separate centrifuge tubes containing the bentonite, and the samples were kept at 25 °C and constantly oscillated at 220 rpm for 24 h.

(2) Effect of Reaction Temperature:

A total of 50 mL of 9 g/L NaCl solution was added to the centrifuge tubes. The tubes were then placed at 10 °C, 20 °C, 30 °C, 40 °C, and 50 °C and oscillated at 220 rpm for 24 h.

The supernatants from the two experiments were filtered using a 0.45 µm water filter, and the Na^+^ concentration was quantified with a flame photometer. The BHWA was then removed, freeze-dried in a freeze-drying oven, and stored for further characterization.

Gibbs free energy equation:

(6) $$\Delta G{}^{\circ}\;=\;-RT\;ln\left(Kd\right)$$

Thermodynamic relationships:

(7) ΔG° = ΔH° − TΔS°

Van ‘t Hoff equation:

(8) $$ln\left(Kd\right)=-\Delta H{}^{\circ}/RT+\Delta S{}^{\circ}/R$$

where ΔG° is the change in Gibbs free energy (J/mol); H° is heat effect (J/mol); ΔS° is the change in system chaos degree (disorder degree) (J/(mol·K)); R is gas constant; 8.314 J/(mol·K); T is absolute temperature (K); Kd = q_e_/C_e_ (mL/g).

#### 4.2.3. Repeated Adsorption–Desorption Test

The 0.25 g BHWA was placed into 100 mL centrifuge tubes, 50 mL of 9 g/L NaCl solution was added, and the tubes were oscillated at 220 rpm and 25 °C for 24 h. Then, the supernatant was filtered through a 0.45 μm water filter and stored. The BHWA was removed, dried in an oven at 60 °C, and returned to the centrifuge tubes. Then, 50 mL of deionized water was added to simulate the desorption process. After shaking for 1 h, the agent was removed, and the supernatant was filtered again through a 0.45 μm water filter. This adsorption–desorption cycle was repeated six times, and the Na^+^ content in the supernatant obtained after each filtration was determined using a flame photometer. After each cycle, the BHWA was freeze-dried and stored for further characterization. Twelve points were selected for EDS analysis on each SEM sample, grouped into three sets of four points, to quantify the weight percentages of Na^+^ and Cl^−^ elements.

#### 4.2.4. Soil Column Experiment

The dried soil was passed through a 2 mm soil sieve to remove coarse particles and small stones, and four layers of gauze were placed on one end of the sieve plate to prevent any soil particles from leaking out. Then, a soil column was filled with the soil at a soil bulk density of 3 g/cm^3^, three acrylic columns were filled with only soil (CK), and the remainder (TB) contained a mixture of soil and BHWA at a BHWA-to-soil ratio of 5:1000. The columns were left to stand for two weeks, and then the bottom of the soil column was placed in a 9 g/L NaCl solution to simulate the migration of groundwater to the soil surface. The soil column test was carried out from 7 November 2024 until the NaCl solution completely reached the top of the soil column on 13 November 2024. The water absorption of the soil column was calculated by recording the amount of water added and the remaining water in the plastic measuring cup after the test.

### 4.3. Instruments

A flame photometer (FP640, Shanghai, China) was used to measure the Na^+^ concentrations in the supernatant after each test. The adsorbed BHWA was then freeze-dried, and the microstructure of the bentonite was characterized using a scanning electron microscope (SEM, AZtecLive Ultim Max 100, Abingdon, UK).

### 4.4. Statistical Analysis

One-way ANOVA was used to analyze the Na^+^ adsorption capacity of BHWA and the Na^+^ weight ratio after complete adsorption. Levene’s test (*p*-value > 0.05) was performed to test the equality of variance assumption. One-way ANOVA was used to compare treatments. The residual distribution and variances were evaluated, which met the ANOVA assumptions. Principal component analysis (PCA) was used to perform linear transformation and dimensionality reduction on the original features (adsorption time, initial concentration of solution, pH, temperature and adsorption frequency).

As described in each illustration, in a completely randomized design, experiments were performed with at least 4 independent replicates. SPSS 25.0 software was used to calculate the analysis of variance. The significance of differences between data sets was evaluated using paired Student’s *t*-test (*p*-value < 0.05). Statistical charts of data, i.e., bar charts, line graphs, principal component load matrix diagrams, and scatter diagrams, were produced using Origin 2021 software.

## Figures and Tables

**Table 1:** Gibbs free energy at various temperatures.

Temperature (°C)	Kd (L/g)	ΔG° (kJ/mol)
10	0.247	+3.29
20	0.759	+0.67
30	1.033	−0.08
40	1.067	−0.17
50	1.243	−0.58

**Table 2:** Principal component analysis matrix.

Module	Component	
	PCA1	PCA2
pH	0.704	0.568
Time	−0.89	0.078
Temperature	0.784	−0.434
Concentration	−0.597	0.662
Frequency	0.645	0.629

## Data Availability

The original contributions presented in this study are included in the article; further inquiries can be directed to the corresponding author.

## Abbreviations

The following abbreviations are used in this manuscript:

BHWA	Bentonite-based hydrogel water-retaining agent
SEM	Scanning electron microscope
PCA	Principal component analysis
